# Naked cuticle homolog 1 prevents mouse pulmonary arterial hypertension via inhibition of Wnt/β-catenin and oxidative stress

**DOI:** 10.18632/aging.205105

**Published:** 2023-10-18

**Authors:** Shanwu Wei, Lu Lin, Wen Jiang, Jie Chen, Gu Gong, Daming Sui

**Affiliations:** 1 Department of Anesthesiology, The General Hospital of Western Theater Command, Chengdu 610083, China; 2 Department of Outpatient, The General Hospital of Western Theater Command, Chengdu 610083, China; 3 Department of Cardiac Surgery, The General Hospital of Western Theater Command, Chengdu 610083, China; 4 Department of Pain Medicine, The General Hospital of Western Theater Command, Chengdu 610083, China

**Keywords:** NKD1, β-catenin, oxidative stress, pulmonary arterial smooth muscle cell, pulmonary hypertension

## Abstract

Pulmonary arterial hypertension (PAH) is a poorly prognostic cardiopulmonary disease characterized by abnormal contraction and remodeling of pulmonary artery (PA). Excessive proliferation and migration of pulmonary arterial smooth muscle cells (PASMCs) are considered as the major etiology of PA remodeling. As a negative regulator of Wnt/β-catenin pathway, naked cuticle homolog 1 (NKD1) is originally involved in the tumor growth and metastasis via affecting the proliferation and migration of different types of cancer cells. However, the effect of NKD1 on PAH development has not been investigated. In the current study, downregulated NKD1 was identified in hypoxia-challenged PASMCs. NKD1 overexpression by adenovirus carrying vector encoding *Nkd1* (Ad-*Nkd1*) repressed hypoxia-induced proliferation and migration of PASMCs. Mechanistically, upregulating NKD1 inhibited excessive reactive oxygen species (ROS) generation and β-catenin expression in PASMCs after hypoxia stimulus. Both inducing ROS and recovering β-catenin expression abolished NKD1-mediated suppression of proliferation and migration in PASMCs. *In vivo*, we also observed decreased expression of NKD1 in dissected PAs of monocrotaline (MCT)-induced PAH model. Upregulating NKD1 by Ad-*Nkd1* transfection attenuated the increase in right ventricular systolic pressure (RVSP), right ventricular hypertrophy index (RVHI), pulmonary vascular wall thickening, and vascular β-catenin expression after MCT treatment. After recovering β-catenin expression by SKL2001, the vascular protection of external expression of NKD1 was also abolished. Taken together, our data suggest that NKD1 inhibits the proliferation, migration of PASMC, and PAH via inhibition of β-catenin and oxidative stress. Thus, targeting NKD1 may provide novel insights into the prevention and treatment of PAH.

## INTRODUCTION

Pulmonary arterial hypertension (PAH) is a kind of cardiopulmonary disease damaging small pulmonary arteries with complicated etiopathogenesis and poor prognosis [1]. The diagnostic criterion for PAH is the fact that resting mean pulmonary artery pressure exceeds 25 mmHg [2]. The canonical pathological characteristics are vascular wall thickening and increased pulmonary vascular resistance, which may eventually evolve to right heart dysfunction, hypertrophy, and even death [3]. Vascular remodeling is a crucial process that drives the development of vascular wall thickening. This complex process includes the endothelial dysfunction, the abnormal proliferation and migration of pulmonary arterial smooth muscle cells (PASMCs), extracellular matrix deposition, and also inflammation [4]. There exists a dynamic balance between proliferation and apoptosis in PASMCs under physiological condition. However, the PASMCs undergo a highly specialized-to-proliferating phenotypic shift when suffering from some pathological stimuli, such as hypoxia [4]. This pathological switching of PASMCs represents an important part of PAH. Although multiple efforts have been made, the clinical outcomes of current therapies focusing on pulmonary artery (PA) vasoconstriction, such as endothelin-1 antagonists, are still unsatisfactory [5, 6]. Thus, targeting proteins involved in the proliferation and migration of PASMCs may represent a breakthrough for preventing or treating PAH.

Originally considered as an antagonist of Wnt signaling, naked cuticle homolog 1 (NKD1) plays a variety of biological roles *in vivo* [7]. The subcellular distribution of NKD1 includes the membrane, the cytoplasm, and also the nucleus [7]. Since dysregulation of NKD1 has been observed in many types of neoplasms, the regulatory roles of NKD1 in cancer are receiving widespread attention. For instance, NKD1 is upregulated in hepatoblastoma and colorectal adenomas whereas NKD1 is downregulated in breast cancer, lung adenocarcinoma, non-small cell lung cancer, and acute myeloid leukemia [8–13]. The modulatory role of NKD1 in cancers largely rely on its regulation of cellular proliferation and migration [14–16]. Nevertheless, whether NKD1 is also involved in the proliferation and migration of PASMCs remains unrevealed.

β-catenin serves as the critical regulator of Wnt signaling pathway and plays an important role in adherens junctions and stabilizing cell-cell contacts [17]. It is demonstrated that increased expression of β-catenin is associated with enhanced proliferation, migration of PASMCs [18], an also accelerated development of PAH [19]. As a negative regulator of Wnt signaling, NKD1 suppresses proliferation in several cancer cell types via reducing β-catenin expression [20, 21]. Oxidative stress is another well-known factor to induce proliferation and migration of PASMCs and is also closely related to Wnt signaling. However, the precise role of β-catenin and oxidative stress in NKD1-mediated regulation of PASMCs and PAH is unclear.

In the current study, we found that the expression of NKD1 was downregulated in hypoxia-challenged PASMCs. Upregulating NKD1 expression by adenovirus carrying vector encoding *Nkd1* (Ad-*Nkd1*) inhibited hypoxia-induced proliferation and migration of PASMCs. In addition, Ad-*Nkd1* transfection also suppressed reactive oxygen species (ROS) generation and β-catenin expression in PASMCs after hypoxia stimulus. Both inducing ROS and recovering β-catenin expression abolished NKD1-mediated repression of proliferation and migration in PASMCs. *In vivo*, by inducing mouse PAH with monocrotaline (MCT), we observed decreased expression of NKD1 in dissected PA. Ad-*Nkd1* transfection attenuated the increase in right ventricular systolic pressure (RVSP), right ventricular hypertrophy index (RVHI), and vascular β-catenin expression after MCT treatment, as well as pulmonary vascular wall thickening. After recovering β-catenin expression, the vascular protection of external expression of NKD1 was also impaired.

## RESULTS

### Effects of upregulating NKD1 on the hypoxia-induced proliferation and migration of PASMCs

**Figure 1:**
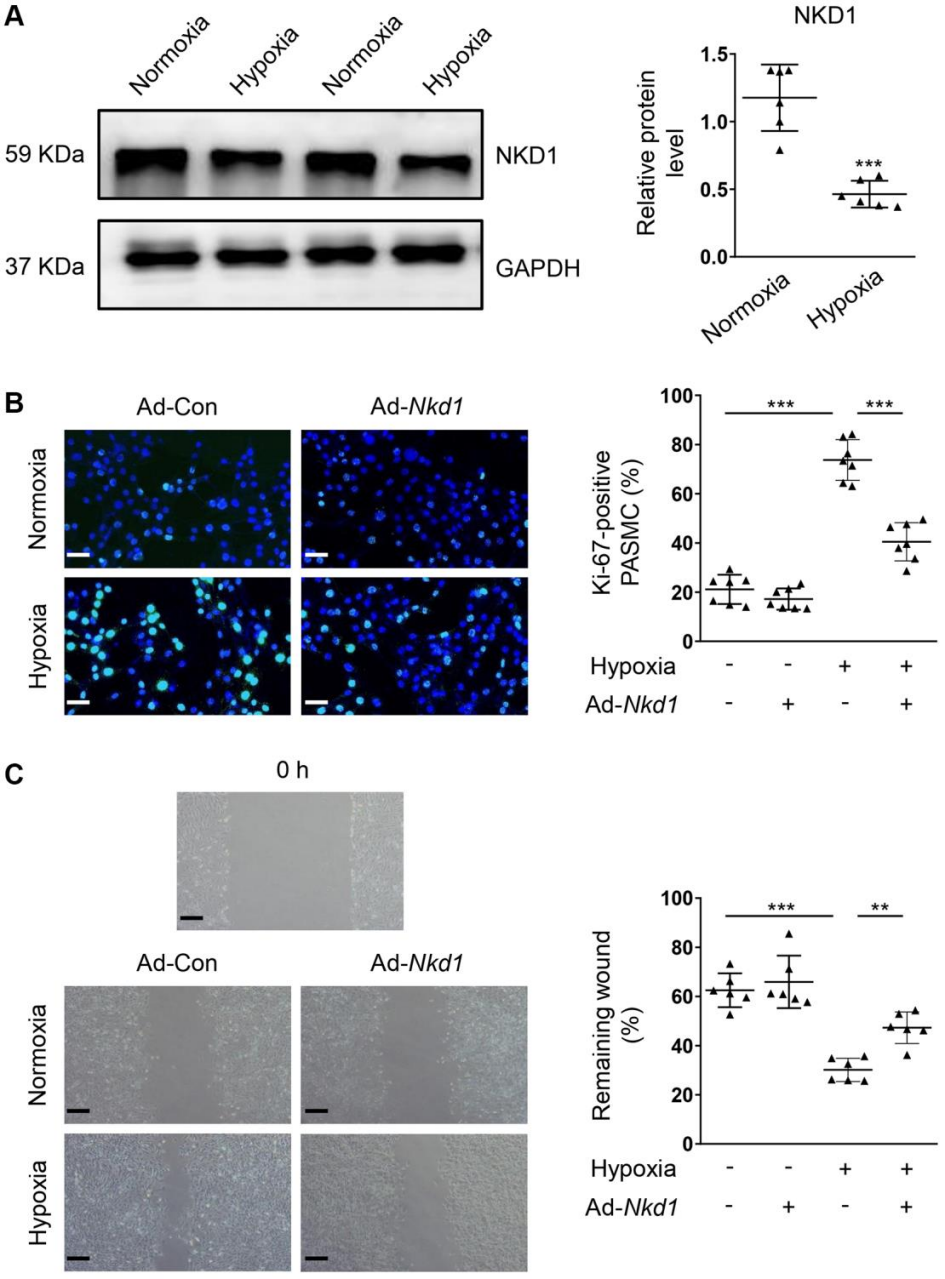
**NKD1 represses the proliferation and migration in hypoxia-treated PASMC.** (**A**) The relative protein expression of NKD1 was assessed by western blotting in PASMCs after 12 h of hypoxia exposure (*post hoc* for LSD test; *n* = 6 samples). PASMCs were transfected with Ad-Con or Ad-*Nkd1* and then cultured in the condition of normoxia or hypoxia. (**B**) Above treated PASMCs were stained with Ki-67 (green) and DAPI (blue). Representative pictures and the corresponding percentage of Ki-67-positive PASMCs were shown (*post hoc* for LSD test; *n* = 7 samples). Bar = 50 μm. (**C**) Migration of above-treated PASMCs were assessed by wound-healing assay (*post hoc* for LSD test; *n* = 6 samples). Bar = 200 μm. Data were shown as mean ± S.D. ^**^P < 0.01 and ^***^P < 0.001 denoted statistical comparison between the two marked groups, respectively.

As an important pathological factor during the development of PAH [3, 22], hypoxia is known to promote the proliferation and migration of PASMCs [4]. In the hypoxia-challenged PASMCs, the protein expression of NKD1 was significantly deceased, compared with that in PASMCs of normoxia group (Figure 1A). To explore the regulatory role of NKD1 in the hypoxia-stimulated PASMCs, we utilized Ad-*Nkd1* transfection to upregulate the NKD1 expression. The external expression of NKD1 was confirmed by western blotting (Supplementary Figure 1). Ki-67 immunofluorescence staining showed that hypoxia stimulus caused increased percentage of Ki-67-positive PASMCs, which was attenuated after upregulating NKD1 expression by Ad-*Nkd1* transfection (Figure 1B). Wound-healing assay was performed to reveal the effect of NKD1 on migration. Similarly, the rate of wound-healing in PASMCs was markedly increased after 12 hours (h) of hypoxia stimulus, whereas NKD1 overexpression significantly reduced the wound-healing rate (Figure 1C). These data suggest that NKD1 suppresses the proliferation and migration of PASMCs after hypoxia challenge.

### NKD1 inhibits hypoxia-induced proliferation and migration of PASMCs through suppressing oxidative stress

**Figure 2:**
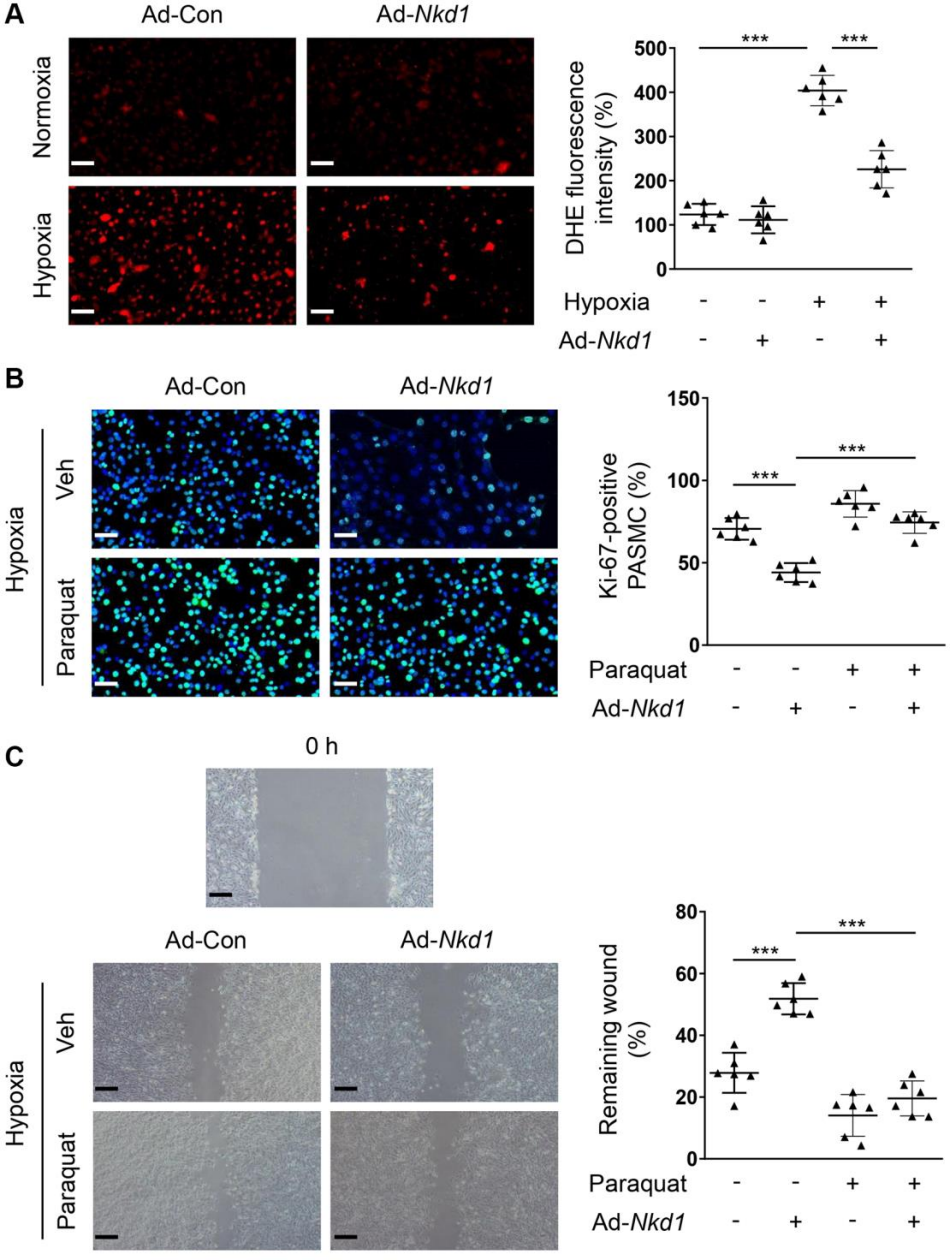
**NKD1 suppresses hypoxia-induced proliferation and migration of PASMCs via inhibiting oxidative stress.** (**A**) PASMCs were transfected with Ad-Con or Ad-*Nkd1* and then cultured in the condition of normoxia or hypoxia. Above treated PASMCs were stained with DHE. Representative pictures and the corresponding quantification of DHE fluorescence intensity were shown (*post hoc* for LSD test; *n* = 6 samples). Bar = 50 μm. Hypoxia-challenged PASMCs were transfected with Ad-Con or Ad-*Nkd1* and then cultured with or without paraquat treatment. (**B**) Above treated PASMCs were stained with Ki-67 (green) and DAPI (blue). Representative pictures and the corresponding percentage of Ki-67-positive PASMCs were shown (*post hoc* for LSD test; *n* = 6 samples). Bar = 50 μm. (**C**) Migration of above-treated PASMCs was assessed by wound-healing assay (*post hoc* for LSD test; *n* = 6 samples). Bar = 200 μm. Data were shown as mean ± S.D. ^***^P < 0.001 denoted statistical comparison between the two marked groups.

Hypoxia-treated PASMCs led to exacerbated generation of ROS, which further promoted the proliferation and migration of PASMCs [23]. Therefore, oxidative stress is an essential mediator of PAH. A significant elevation in the dihydroethidium (DHE) fluorescence intensity was observed when PASMCs were treated in hypoxic condition in the current study. Next, we transferred PASMCs with Ad-*Nkd1* and found the intensity of DHE was obviously attenuated (Figure 2A), indicating that NKD1 suppressed ROS generation in hypoxia-challenged PASMCs. To verify that the downregulation of ROS generation was responsible for NKD1-mediated inhibition of proliferation and migration in PASMCs, we used a ROS inducer, paraquat [24] to induce oxidative stress. After application of paraquat, the decrease in the percentage of Ki-67-positive PASMCs and the reduced rate of wound-healing induced by upregulating NKD1 were both reversed (Figure 2B, 2C). Thus, we demonstrated that NKD1 represses hypoxia-induced proliferation and migration of PASMCs via reducing oxidative stress.

### NKD1 suppresses oxidative stress in PASMCs after hypoxic stimulus via reducing β-catenin expression

**Figure 3:**
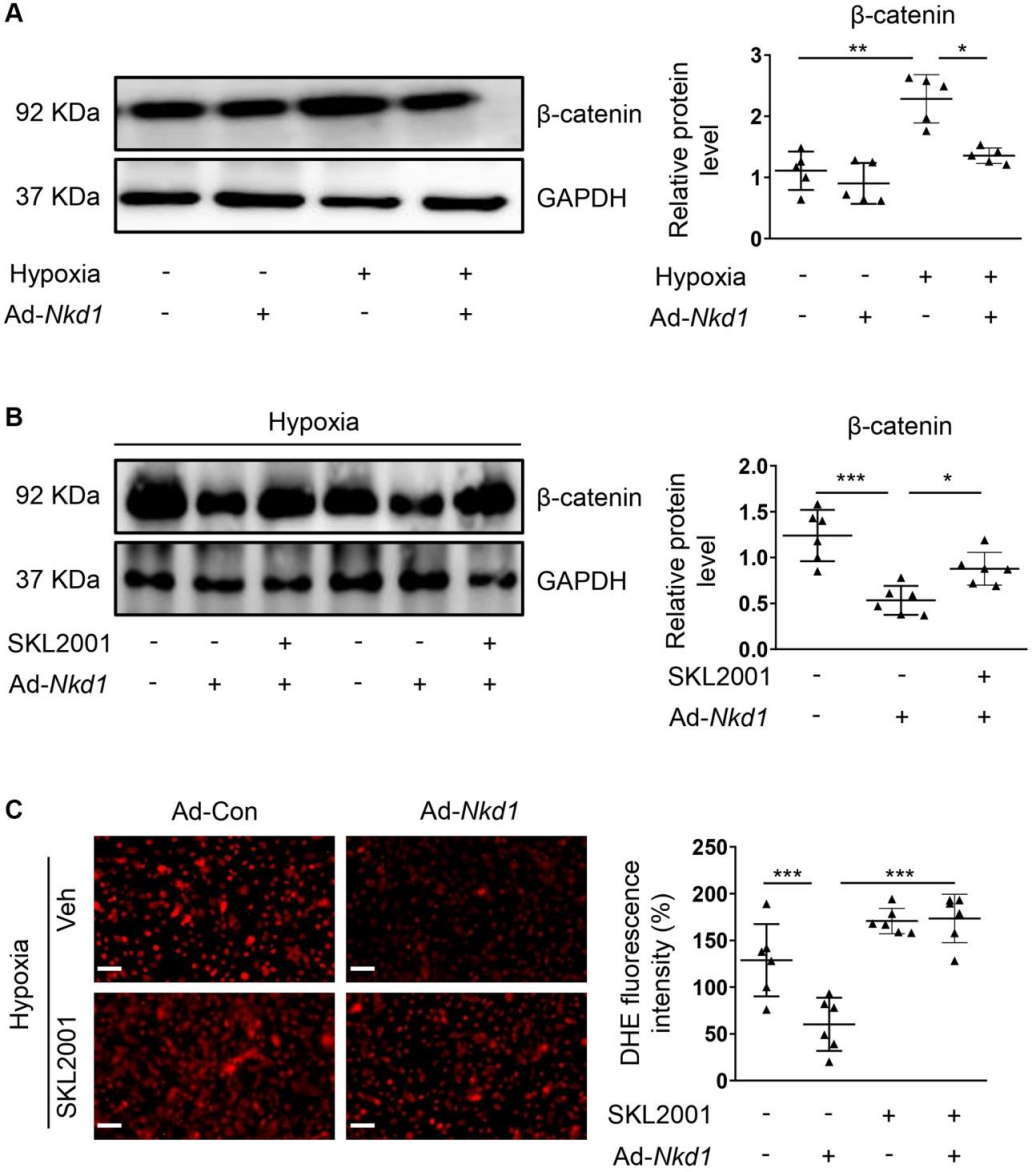
**Downregulated β-catenin is responsible for NKD1-mediated regulation of oxidative stress in hypoxia-treated PASMCs.** (**A**) PASMCs were transfected with Ad-Con or Ad-*Nkd1* and then cultured in the condition of normoxia or hypoxia. The relative expression of β-catenin in above-treated PASMCs was assessed by western blotting (*post hoc* for Dunnett’s T3 test; *n* = 5 samples). Hypoxia-challenged PASMCs were transfected with Ad-Con or Ad-*Nkd1* and then cultured with or without SKL2001 treatment. (**B**) The relative expression of β-catenin in above-treated PASMCs was assessed by western blotting (*post hoc* for LSD test; *n* = 6 samples). (**C**) Above treated PASMCs were stained with DHE. Representative pictures and the corresponding quantification of DHE fluorescence intensity were shown (*post hoc* for LSD test; *n* = 6 samples). Bar = 50 μm. Data were shown as mean ± S.D. ^*^P < 0.05, ^**^P < 0.01 and ^***^P < 0.001 denoted statistical comparison between the two marked groups, respectively.

**Figure 4:**
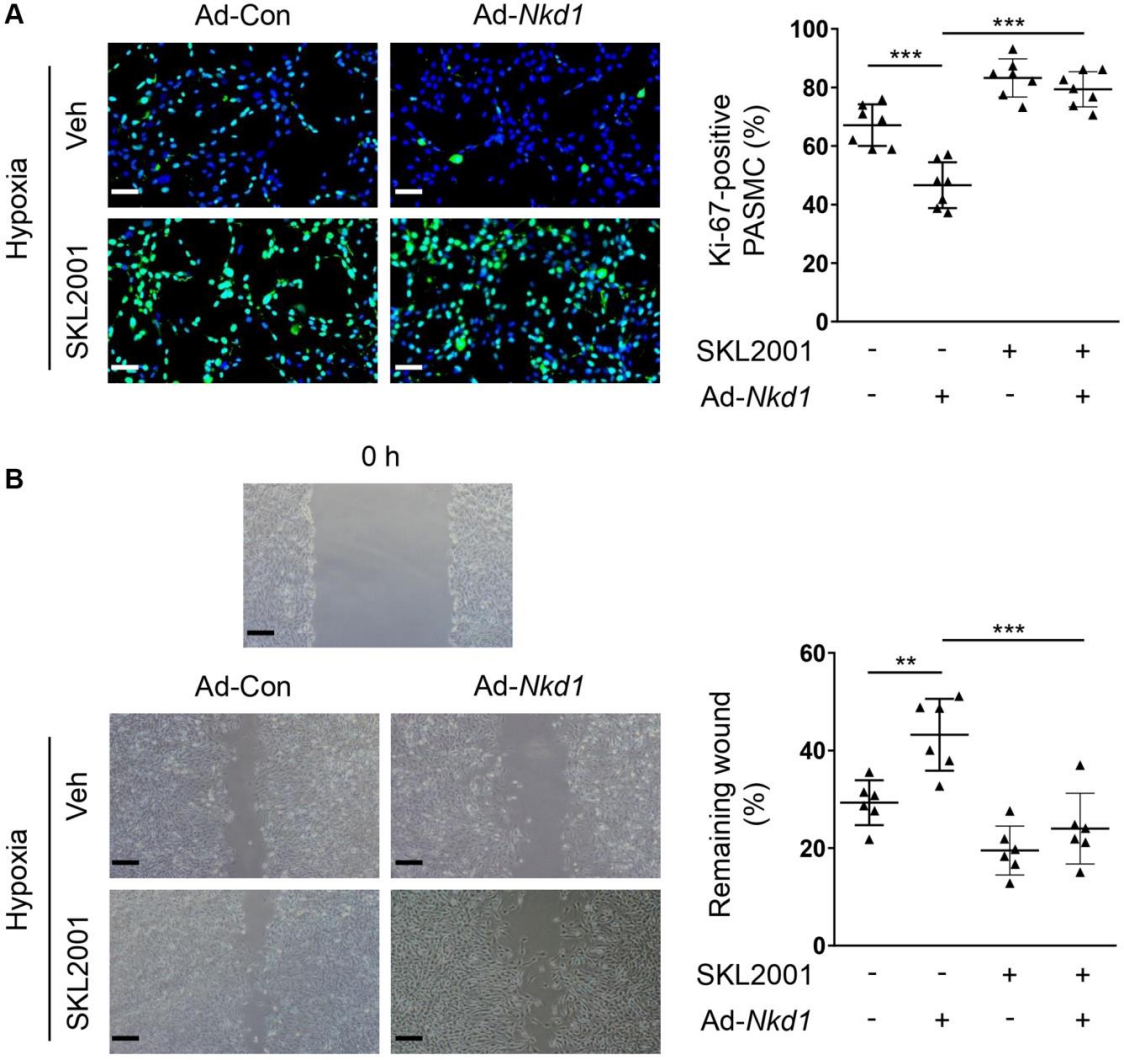
**NKD1 inhibits hypoxia-induced proliferation and migration of PASMCs via reducing β-catenin expression.** Hypoxia-challenged PASMCs were transfected with Ad-Con or Ad-*Nkd1* and then cultured with or without SKL2001 treatment. (**A**) Above treated PASMCs were stained with Ki-67 (green) and DAPI (blue). Representative pictures and the corresponding percentage of Ki-67-positive PASMCs were shown (*post hoc* for LSD test; *n* = 7 samples). Bar = 50 μm. (**B**) Migration of above-treated PASMCs were assessed by wound-healing assay (*post hoc* for LSD test; *n* = 6 samples). Bar = 200 μm. Data were shown as mean ± S.D. ^**^P < 0.01 and ^***^P < 0.001 denoted statistical comparison between the two marked groups, respectively.

NKD1 is known to negatively modulate β-catenin, which is a crucial regulator of proliferation and migration in PASMCs [7, 25]. Additionally, β-catenin is also confirmed to aggravate oxidative stress-induced injury [26]. To investigate whether β-catenin was involved in NKD1-mediated regulation of oxidative stress, we firstly examined the expression of β-catenin in hypoxia-treated PASMCs after Ad-*Nkd1* transfection. As expected, the protein level of β-catenin was significantly augmented in hypoxia-challenged PASMCs. Whereas, upregulating NKD1 markedly attenuated the hypoxia-induced expression of β-catenin (Figure 3A). Then, we utilized an agonist of β-catenin named SKL2001 [27] to recover the reduced expression of β-catenin induced by NKD1 overexpression. Pretreatment of SKL2001 rescued the decreased β-catenin expression in hypoxia-challenged PASMCs transfected with Ad-*Nkd1* (Figure 3B). As DHE immunofluorescence staining showed, SKL2001 markedly elevated the ROS generation in hypoxia-challenged PASMCs after upregulating NKD1 (Figure 3C). Notably, after application of SKL2001, the attenuated proliferation and migration of hypoxia-challenged PASMCs induced by upregulating NKD1 were also abolished (Figure 4A, 4B). Thus, these results indicate that β-catenin is involved in NKD1-mediated regulation of oxidative stress, proliferation, and migration in PASMCs.

### Upregulating NKD1 prevents against PAH *in vivo*

**Figure 5:**
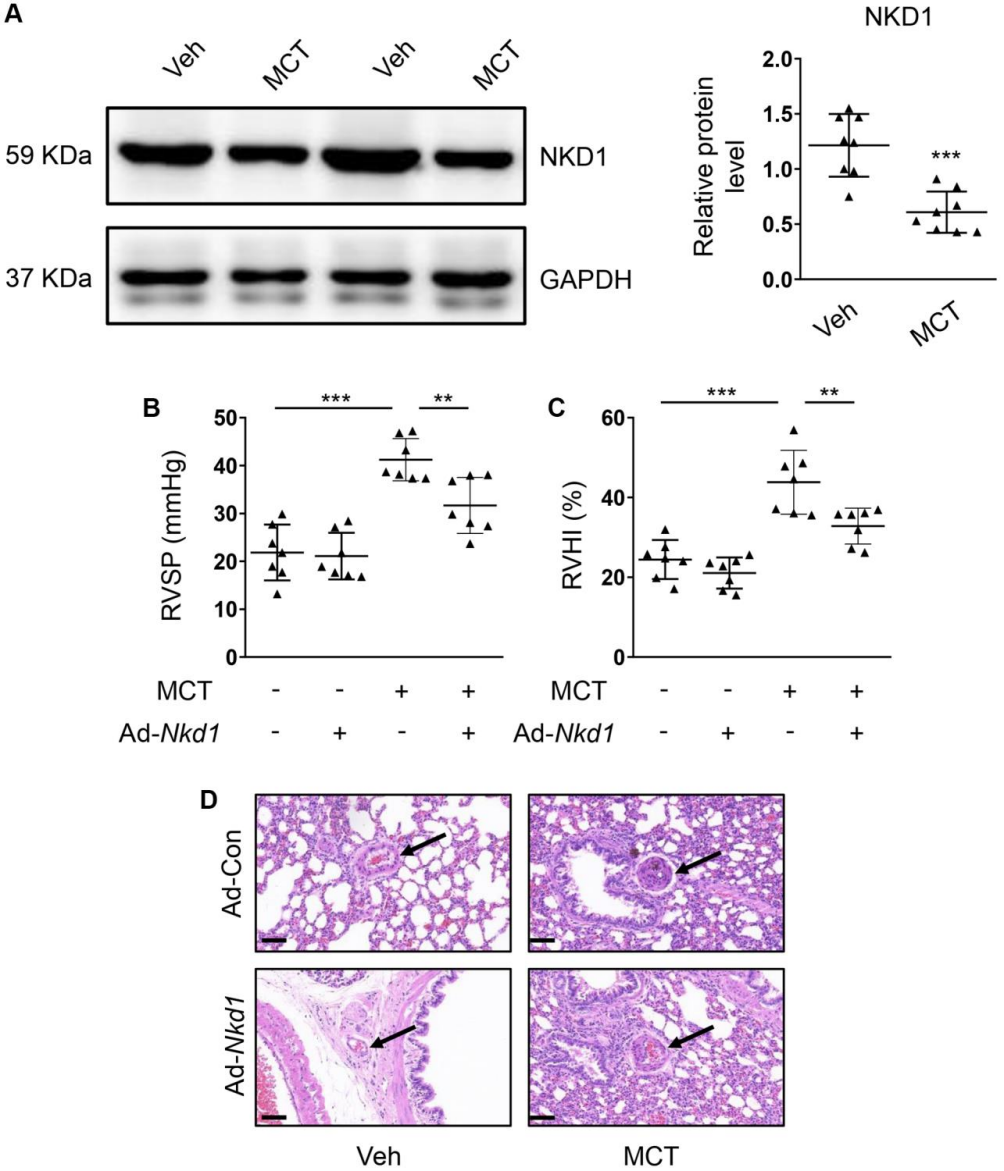
**Upregulating NKD1 alleviates PAH symptoms in MCT-induced mouse PAH model.** (**A**) The relative protein level of NKD1 in dissected PAs of MCT-treated mice was assessed by western blotting (*post hoc* for LSD test; *n* = 8 samples). RVSP (**B**) and RVHI (**C**) of MCT-treated mice were measured (*post hoc* for LSD test; *n* = 7 samples). RVSP indicates right ventricular systolic pressure. RVHI indicates right ventricular hypertrophy index. (**D**) Representative H-E staining pictures of lung tissues in vehicle- or MCT-treated mice after transfection of Ad-Con or Ad-*Nkd1* were shown. The black arrows indicate peripheral PAs of lung tissues. Bar = 50 μm. Data were shown as mean ± S.D. ^**^P < 0.01 and ^***^P < 0.001 denoted statistical comparison between the two marked groups, respectively.

Upregulating NKD1 could inhibit the proliferation and migration of PASMCs in the hypoxia condition. Therefore, we also evaluated the therapeutic potential of NKD1 in MCT-induced mouse PAH model *in vivo*. By using MCT administration, the RVSP and RVHI in mouse were both obviously increased (Supplementary Figure 2A, 2B), which meant that the MCT-induced PAH model was successfully established. Simultaneously, the expression of NKD1 was also downregulated in PA of MCT-induced mouse PAH model (Figure 5A). After transfection of Ad-*Nkd1 in vivo*, we found that the expression of NKD1 was markedly augmented in PA (Supplementary Figure 2C). Treatment with Ad-*Nkd1* transfection obviously ameliorated the elevated RVSP and RVHI in MCT-induced mouse PAH model (Figure 5B, 5C). Hematoxylin-eosin (H-E) staining was performed to investigate the role of NKD1 in PA morphology. As the result showed, mice of MCT group displayed accelerated vascular wall thickening and intimal hyperplasia. Whereas, these pathological changes were markedly ameliorated by upregulating NKD1 expression (Figure 5D). Notably, despite that NKD1 partly alleviated the development of PAH, these parameters could not return to normal. Thus, we demonstrated that NKD1 also played a protective role in PAH *in vivo*.

### NKD1 alleviates mouse PAH by inhibiting the vascular expression of β-catenin

**Figure 6:**
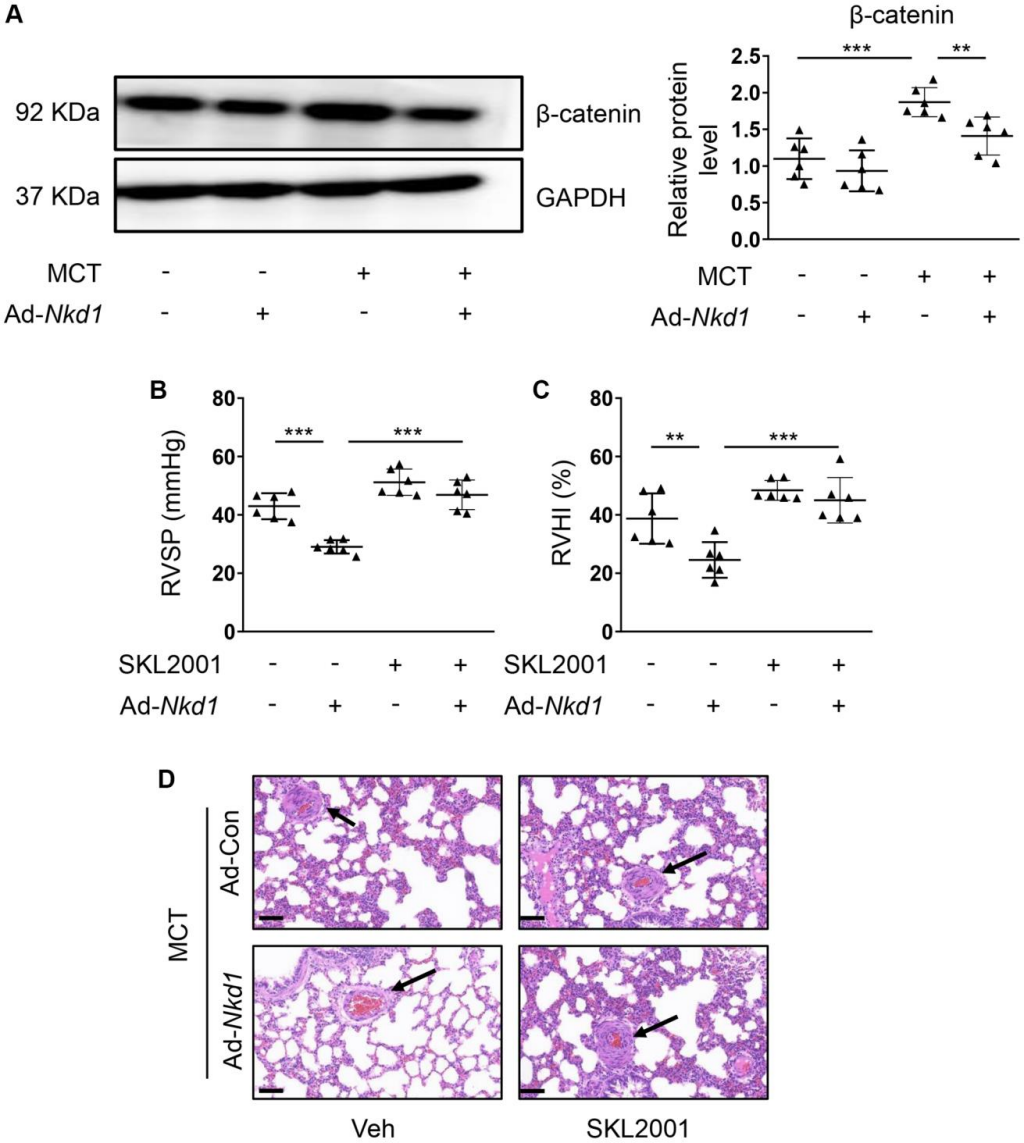
**β-catenin is required for NKD1-mediated protection against PAH.** (**A**) The relative protein level of β-catenin in dissected PAs of MCT-treated mice after transfection of Ad-Con or Ad-*Nkd1* was assessed by western blotting (*post hoc* for LSD test; *n* = 6 samples). MCT-treated mice were transfected with Ad-Con or Ad-*Nkd1* and treated with or without SKL2001. RVSP (**B**) and RVHI (**C**) of above-treated mice were measured (*post hoc* for LSD test; *n* = 6 samples). (**D**) Representative H-E staining pictures of lung tissues in above-treated mice were shown. The black arrows indicate peripheral PAs of lung tissues. Bar = 50 μm. Data were shown as mean ± S.D. ^**^P < 0.01 and ^***^P < 0.001 denoted statistical comparison between the two marked groups, respectively.

β-catenin is also involved in the development of PAH [28]. In the current study, an obvious elevated expression of β-catenin in PA was found in MCT-treated mouse. Furthermore, the increased β-catenin expression in MCT-induced PAH model was downregulated after upregulating NKD1 by Ad-*Nkd1* (Figure 6A). This reminded us that β-catenin might also participate in NKD1-mediated protection against PAH. Next, we used SKL2001 [29] *in vivo* to rescue the attenuated β-catenin expression induced by NKD1 overexpression. As expected, declined RVSP and RVHI after upregulating NKD1 were both augmented by pretreatment of SKL2001 (Figure 6B, 6C). H-E staining further revealed that the attenuated vascular wall thickening and intimal hyperplasia in NKD1-overexpressing PA were remarkably reversed after SKL2001 treatment (Figure 6D). Moreover, we also measured the levels of enzymes that were closely related to modulation of oxidative stress. The malondialdehyde (MDA) level was significantly decreased while the levels of antioxidant superoxide dismutase (SOD) and glutathione (GSH) were increased after upregulating NKD1 in PAs of MCT-treated mice (Supplementary Figure 3), suggesting that NKD1 could alleviate oxidative stress in mouse PAH model. After application of SKL2001, the inhibitory effect of NKD1 on oxidative stress *in vivo* disappeared (Supplementary Figure 3). We thus confirmed that NKD1 suppressed mouse PAH development and oxidative stress via inhibition of β-catenin.

## DISCUSSION

PAH is a cardiopulmonary disease that has poor prognosis and complicated etiopathogenesis. The abnormal proliferation and migration of PASMCs constitute the major step of PAH. However, the clinical effects of current therapeutic methods focusing on PAH were still not satisfactory. In the current study, we identified the downregulation of NKD1 in the hypoxia-challenged PASMCs. Upregulating NKD1 evidently suppressed the hypoxia-induced proliferation and migration of PASMCs. We further determined that NKD1 inhibited ROS generation and β-catenin expression in PASMCs after hypoxia stimulus. In addition, both ROS inducer and β-catenin agonist abolished the suppressive effects of NKD1 on the hypoxia-induced proliferation and migration of PASMCs. *In vivo* studies demonstrated that NKD1 expression was significantly downregulated in PA of MCT-induced mouse PAH model. Upregulating NKD1 ameliorated elevated RVSP, RVHI, vascular intimal hyperplasia of PA, and oxidative stress in MCT-treated mice. Notably, the preventive roles of NKD1 in PAH were also abolished after application of β-catenin agonist. Thus, NKD1 may be considered as a novel potent target to prevent PAH.

Originally observed in Drosophila, NKD1 is widely expressed in a variety of organs and tissues. There are four conserved domains in NKD protein family, including NH1, NH2, NH3, and NH4 domains [30]. Due to the special protein domains, NKD1 is able to directly regulate gene transcription [31]. Since the expression of NKD1 is dysregulated in a series of cancers, NKD1 is considered as an important regulator or predictor of tumor progression [13–15, 32]. The regulation of NKD1 on tumor progression is biphasic, resulting from the biphasic modulatory effects of NKD1 on proliferation and migration of different tumor cells. For example, NKD1 enhances the proliferation and migration of colon cancer cells [14] while inhibits hepatocellular carcinoma cell proliferation [33]. In our current study, we also observed abundantly expressed NKD1 in PASMCs. The decreased expression of NKD1 was associated with enhanced proliferation and migration of PASMCs after hypoxia stimulus, indicating a potential mechanistic role of NKD1 in PASMCs. By confirming the upregulated expression of NKD1 in PASMCs after Ad-*Nkd1* transfection, hypoxia-induced proliferation and migration in PASMCs were both attenuated. Overall, these findings indicate that NKD1 may contribute to the antiproliferative and anti-migrative phenotype that characterizes PAH.

Aggravated oxidative stress is associated with hypoxia-induced proliferation and migration of PASMCs [34]. Suppressing excessive ROS generation is essential for preventing abnormal proliferation and migration of PASMCs [35]. Indeed, we also observed significantly enhanced ROS generation in hypoxia-treated PASMCs in the current study. Whereas, no reports in the literature to date have raised that NKD1 affects oxidative stress in PASMCs. The current results revealed that hypoxia-induced excessive ROS generation was attenuated by upregulating NKD1. We also revealed that the NKD1-mediated inhibitory effects of NKD1 on hypoxia-induced proliferation and migration in PASMCs were also abolished after inducing ROS by paraquat. Therefore, oxidative stress may act as a crucial factor involved in NKD1-mediated inhibition of proliferation and migration in hypoxia-challenged PASMCs.

NKD1 can bind Dishevelled and subsequently suppress β-catenin nuclear localization and transcriptional activation, which finally leads to decreased β-catenin expression [36]. As a canonical effector of Wnt signaling pathway, β-catenin can directly regulate the transcription of target genes which affect cell proliferation and survival [37]. There are also several lines of evidence that β-catenin also promotes the proliferation and migration of PASMCs [18, 19]. However, whether NKD1 regulates β-catenin expression in PASMCs is still unrevealed. In the current study, β-catenin expression was upregulated after hypoxia stimulus, while the increased expression of β-catenin was attenuated after Ad-*Nkd1* transfection. Previous studies demonstrate that NKD1 can inhibit proliferation and migration in several cancer cell lines via inhibiting β-catenin [20, 21]. Consistently, we also revealed that β-catenin agonist, SKL2001 also abrogated NKD1-mediated inhibition of proliferation and migration in hypoxia-treated PASMCs. Additionally, increased β-catenin expression is also associated with accelerated oxidative stress in heart [26]. Whether this relationship also exists in PASMCs is unclear. Our *in vitro* experiments further confirmed that the attenuated ROS generation induced by upregulating NKD1 was reversed after application of SKL2001. These data suggest that NKD1 inhibits the proliferation, migration, and oxidative stress in hypoxia-challenged PASMCs in a β-catenin-dependent manner.

In this study, the expression of NKD1 in PA was downregulated in a validated mouse PAH model [38], and upregulating NKD1 expression by Ad-*Nkd1* transfection could suppress the hypoxia-induced proliferation and migration of PASMCs. Therefore, NKD1 might be involved in the pathogenesis of PAH. Interestingly, we observed that upregulating NKD1 expression could ameliorate PAH-associated pathological changes in MCT-treated mice, as evidenced by the decreased RVSP, RVHI, and attenuated intimal hyperplasia in PA. We also determined that β-catenin expression in PA was also inhibited by upregulating NKD1. More importantly, NKD1-mediated suppression of RVSP, RVHI, and vascular intimal hyperplasia in MCT-induced PAH model were both abrogated after recovering β-catenin expression by SKL2001. Consistent with prior published studies [39, 40], the current study revealed that oxidative stress, characterized by downregulated levels of SOD, GSH while increased MDA level, was aggravated in PA of MCT-treated mice, which was then attenuated after upregulating NKD1. Furthermore, NKD1-mediated regulation of oxidative stress in PAH was abolished after the application of SKL2001. Thus, we propose that NKD1 might be a novel therapeutic target for preventing PAH via inhibiting β-catenin and oxidative stress.

In conclusion, this study demonstrated that NKD1 was downregulated in hypoxia-treated PASMCs and PA of MCT-induced mouse PAH model. Upregulating NKD1 protected from PAH and repressed the hypoxia-induced proliferation and migration of PASMCs. Furthermore, NKD1 acted upstream of β-catenin and oxidative stress during PAH development. Collectively, NKD1 could be taken as a viable therapeutic target for treating PAH by focusing on β-catenin and oxidative stress.

## MATERIALS AND METHODS

### Primary PASMCs culture

Mouse primary PASMCs were isolated from PAs of 10- to 12-week-old C57BL/6J mice by using an enzymatic dissociation method as a previous study described [41]. The isolated PASMCs were cultured with Dulbecco’s Modified Eagle’s Medium (DMEM) supplied with 15% fetal bovine serum (FBS) and 1% antibiotics (Gibco, CA, USA) at 37°C. PASMCs in normaxia group were incubated in a humidified incubator with 21% O_2_, 74% N_2_ and 5% CO_2_. PASMCs in hypoxia group were incubated in a humidified incubator with 3% O_2_, 92% N_2_ and 5% CO_2_. For adenovirus transfection, Ad-*Nkd1* (3 pfu/cell; Genechem, Shanghai, China) or the corresponding adenovirus carrying control empty vector (Ad-Con; 3 pfu/cell; Genechem) was incubated with PASMCs for 48 h changing the medium. For paraquat treatment, PASMCs were treated with paraquat (500 μmoL; MCE, Shanghai, China) for 1 h [24]. For SKL2001 treatment, PASMCs were treated with SKL2001 (40 μmol/L; MCE) for 6 h [27].

### Western blotting

PAs dissected from lung tissue and PASMCs were lysed with RIPA buffer (Beyotime Institute of Biotechnology, Shanghai, China) to extract the total protein. 10 μg of protein sample was separated by sodium dodecyl sulfate-polyacrylamide gels and subsequently transferred to polyvinylidene fluoride membranes (Millipore, MA, USA), blocked by 5% bovine serum albumin for 1 h at room temperature, and then incubated overnight with corresponding primary antibodies at 4°C. Finally, the membranes carrying protein lanes were incubated for 1 h with corresponding secondary antibodies at room temperature before exposure. The primary antibodies against NKD1, β-catenin, and glyceraldehyde-3-phosphate dehydrogenase (GAPDH) were purchased from Cell Signaling Technology (MA, USA).

### Immunofluorescence staining

The proliferation of PASMCs was determined by using Ki-67 immunofluorescence staining as a previous study described [42]. Briefly, PASMCs were cultured in a 6-well plate which has a slide at the bottom of per well. After transfection of corresponding adenovirus or treatments, cells were fixed for 20 min with 4% paraformaldehyde, permeabilized with Triton X-100 for 15 min. Then, cell slides were incubated with the Ki-67 antibody (1: 1000; Cell Signaling Technology) at 4°C in dark. After the incubation overnight, the slides were then incubated with Alexa Fluor 488-conjugated goat anti-rabbit (1: 3000; Molecular Probes Inc., OR, USA) for 1 h in dark. Finally, the slides were incubated with 4′,6-diamidino2-phenylindole (DAPI) (5 mg/ml; Vector Labs, CA, USA) for 5 seconds (s) at room temperature. Representative images were captured under an immunofluorescent microscopy (Leica MPS 60; HD).

DHE staining was performed as a previous study described [43]. Briefly, PASMCs were incubated at 37°C with DHE (5 μmol/L; Beyotime Institute of Biotechnology) for 45 min and then washed by Hank’s balanced salt solution (HBSS; Invitrogen, MA, USA). Finally, representative images were captured under an immunofluorescent microscopy (Leica MPS 60).

### Wound-healing assay

A scratch assay as a previous study described [44] was performed to analyze the migration of PASMCs. PASMCs were seeded in a 6-well plate until the cell density has grown to 90%. Subsequently, a straight scratch was made in the cells by using a 200-μL pipette tip. PASMCs were then cultured for further 12 h after corresponding treatments. Images were captured at 0 and 12 h by using an optical microscope. Cell migration was assessed as the percentage of the remaining wound.

### Animal experiments

The C57BL/6J mice in this study were obtained from Dashuo Animal Science and Technology (Sichuan, China). All animal experiments were performed in accordance with the Institutional Animal Care and Use Committee and the Ethic Committee of The General Hospital of Western Theater Command (Sichuan, China). All mice were housed with a 12-hour light/dark cycle, periodic air changes, and free access to water and food. MCT-induced mouse PAH model was conducted as a previous study described [38]. Briefly, 10- to 12-week-old mice weekly received subcutaneous injection of MCT (60 mg/kg; Sigma) for consecutive 4 weeks. Ad-*Nkd1* (10^11^ pfu/mL) or Ad-Con was weekly injected via the tail vein.

For hemodynamic measurement, mice were firstly anesthetized with pentobarbital (30 mg/kg) via intraperitoneal injection. A pressure transducer catheter (Millar Instruments, TX, USA) was used to determine RVSP. Mice were finally anesthetized with pentobarbital (100 mg/kg) and sacrificed to remove the heart and lung tissues. RVHI was calculated as the percentage ratio of right ventricle/(septum + left ventricle).

### H-E staining

The lung tissues were collected, fixed with 4% formaldehyde, embedded and cut into 5-μm slices. Subsequently, slices were stained with hematoxylin and eosin as a previous study performed [45]. The morphology was analyzed under a microscopy.

### Assessment of oxidative stress markers

The dissected PAs from mice were used to measure the oxidative status by analyzing the levels of SOD, GSH, and MDA as a previous study described [46]. These markers were measured by using ELISA kits (Solarbio, Shanghai, China) according to the manufacturer’s instructions.

### Statistical analysis

All data were presented as mean ± S.D. All statistical analyses were performed by using SPSS 22.0 software. All experiments in this study were repeated for at least 3 times. Unpaired Student’s *t*-test was used between two independent groups. One-way analysis of variance (ANOVA) was performed for multi-comparisons with appropriate *post hoc* tests. *P* value < 0.05 was considered statistically significant.

## Supplementary Materials

Supplementary Figures
