# Supplementary material for: Naked cuticle homolog 1 prevents mouse pulmonary arterial hypertension via inhibition of Wnt/β-catenin and oxidative stress
Source: Aging (Albany NY). 2023 Oct 18;15(20):11114–30. doi: 10.18632/aging.205105 (PMC10637826; doi:10.18632/aging.205105)
Supplement: Supplementary Figures [file aging-15-205105-s001.pdf]

## SUPPLEMENTARY FIGURES

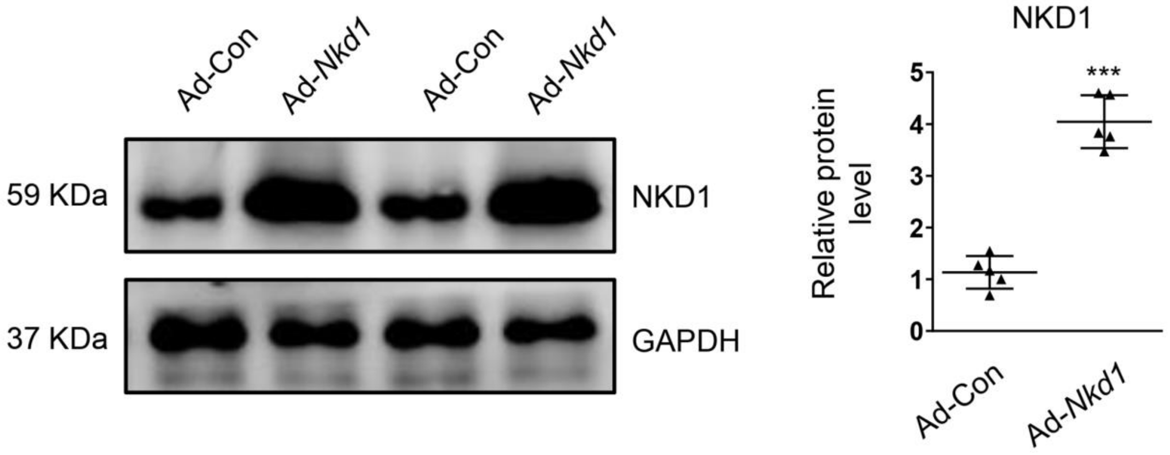


**Supplementary Figure 1. Ad-*Nkd1* transfection increases NKD1 expression in PASMCs.** PASMCs were transfected with Ad-Con or Ad-*Nkd1*. The relative protein expression of NKD1 was assessed by western blotting (*post hoc* for LSD test; *n* = 5 samples). Data were shown as mean ± S.D. $^{***}P < 0.001$ denoted statistical comparison between the two marked groups.

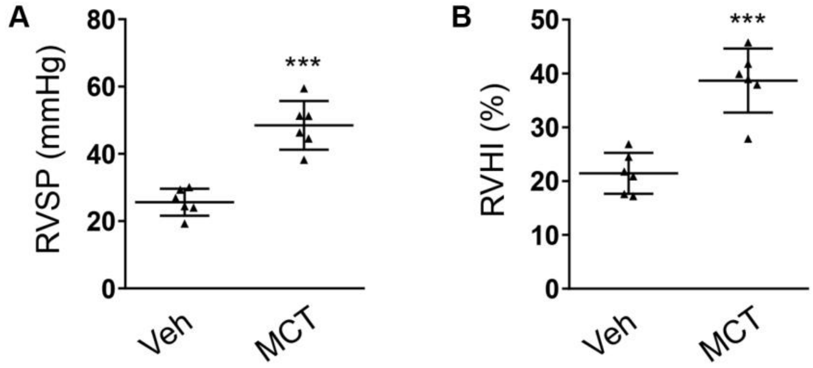


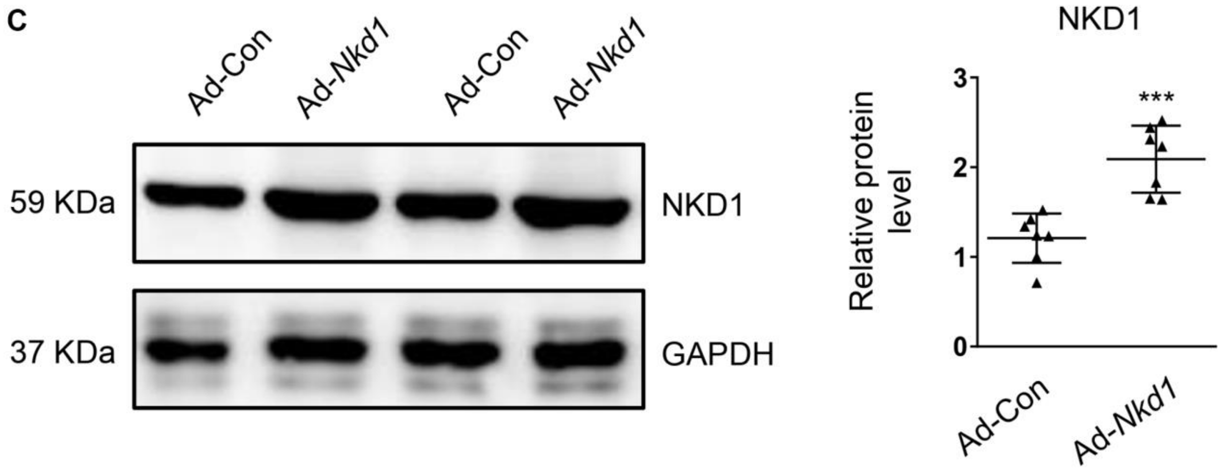


**Supplementary Figure 2. Validation of MCT-induced mouse PAH model establishment and Ad-*Nkd1* transfection efficiency.** RVSP (**A**) and RVHI (**B**) of MCT-treated mice were measured (*post hoc* for LSD test; *n* = 6 samples). (**C**) Mice were transfected with Ad-Con or Ad-*Nkd1*. The relative protein expression of NKD1 was assessed by western blotting (*post hoc* for LSD test; *n* = 7 samples). Data were shown as mean ± S.D. $^{***}P < 0.001$ denoted statistical comparison between the two marked groups.

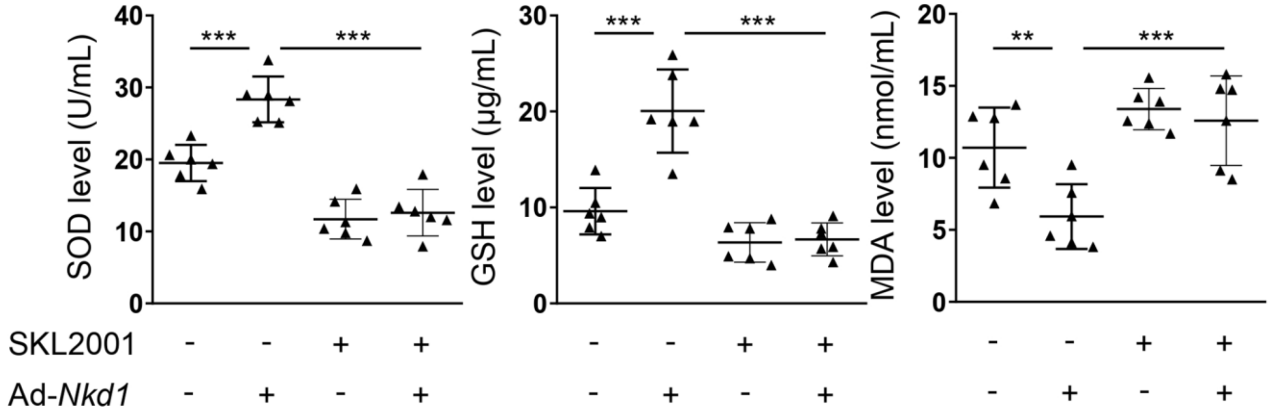


**Supplementary Figure 3. NKD1 suppresses oxidative stress in PAs of MCT-treated mice via reducing β-catenin expression.** The levels of SOD, GSH, and MDA in PAs from different groups were analyzed by ELISA (*post hoc* for LSD test; *n* = 6 samples). Data were shown as mean ± S.D. $^{**}P < 0.01$ and $^{***}P < 0.001$ denoted statistical comparison between the two marked groups, respectively.
